# Study on Performance of Retarded Composite Semi-Rigid Base Mixed with Rubber Powder

**DOI:** 10.3390/ma15134683

**Published:** 2022-07-04

**Authors:** Zhenxia Li, Tengteng Guo, Yuanzhao Chen, Yunpeng Wang, Yanyan Chen, Qingyun He, Xiao Yang, Jing Wang

**Affiliations:** 1 School of Civil Engineering and Communication, North China University of Water Resources and Electric Power, Zhengzhou 450045, China; zhenxiali2009@ncwu.edu.cn (Z.L.); guotth@ncwu.edu.cn (T.G.); wyp0053@163.com (Y.W.); cyy05062022@163.com (Y.C.); 18236567795@163.com (Q.H.); sendeyouxiang@163.com (X.Y.); wj@ncwu.edu.cn (J.W.); 2 Henan Province Engineering Technology Research Center of Environment Friendly and High-Performance Pavement Materials, Zhengzhou 450045, China; 3 Zhengzhou City Key Laboratory of Environmentally Friendly High Performance Road and Bridge Materials, Zhengzhou 450045, China

**Keywords:** road engineering, rubber powder, micro-analysis, semi-rigid base, retarder

## Abstract

In order to solve the problem of poor crack resistance and frost resistance of semi-rigid base, rubber powder and retarder were added to a semi-rigid base mixture. First, 61 mixing ratios were determined. Then, through the unconfined compressive strength, splitting strength, and other tests, the mechanical, crack, and frost resistance properties of the retarded composite semi-rigid base coarse mixture with rubber powder were studied. Finally, the macro and micro properties of the two kinds of admixture composite semi-rigid base coarse mixtures were studied by means of SEM and industrial CT. The results show that rubber powder and retarder can effectively improve the cracking and freezing resistance of the mixture. After five freeze–thaw cycle tests, the strength of the retarded composite semi-rigid base material mixed with rubber powder decreased slightly compared with the mixture without additives. It can be seen that rubber powder improved the frost resistance of the mixture. When the content of rubber powder was 1.5%, the BDR value of the mixture increased by 8.8%. With the increase of unconfined compressive strength, splitting strength, and flexural tensile strength at 28 d and 90 d, it was found that the retarder improved the middle and late strength of the mixture. When the content of retarder was 0.09%, the increase of unconfined compressive strength at 28 d reached 3.9%. The addition of rubber powder and retarder improved the distribution of internal pores, the proportion of large pores decreased, and the proportion of small pores increased. The retarder changed the morphology of hydration products, formed a dense network supporting structure, further refined the pores, and reduced the porosity of the mixture. The proportion of pores with a volume greater than 100 mm^3^ in the total pore volume decreased by 26.01%, and the proportion of medium pores increased by 13.07%, thereby improving the mechanical properties of the mixture.

## 1. Introduction

At present, China is the largest producer of waste tires. The output of waste tires is nearly 400 million, and the growth rate is steadily rising at 8–10% [1,2,3,4]. How to effectively and reasonably recycle them is a global technical problem and an environmental protection problem. If the waste tires are crushed and applied to cement-stabilized macadam base, asphalt mixture, and other road projects, it will not only solve the problem of waste tires, but also save resources. China’s highway construction is mainly based on cement-stabilized macadam semi-rigid base, which has advantages of good integrity and simple construction technology. However, its main defect is that it is easy to produce shrinkage cracks [5,6], thus endangering the integrity and strength of the base, and seriously damaging the pavement performance and life [7,8]. Therefore, in order to solve the problems of waste tires and base cracking, it is necessary to improve the road performance of the base.

Relevant studies have shown that rubber powder can significantly improve the crack resistance of cement concrete, while the deformability, toughness, ductility, carbonation resistance, and water absorption resistance are enhanced [9,10,11]. However, the compressive strength, splitting strength, and flexural tensile strength of cement concrete with waste rubber powder decreased to some extent [9,10,12]. A comprehensive study of the performance of concrete with rubber replacing part of coarse aggregate showed that the increase of rubber content reduced the compressive strength of concrete [13,14,15,16]. The incorporation of rubber particles reduced the 7 d unconfined compressive strength of cement-stabilized macadam, and the greater the dosage, the greater the reduction [17]. The compressive strength and splitting strength of cement-stabilized macadam are negatively correlated with the content of rubber, and the loss rate of compressive strength and splitting strength are positively correlated with the particle size of rubber [18,19]. The cement-stabilized macadam mixed with 0.5% rubber particles has good frost resistance, and the dry shrinkage strain increases slowly, which is conducive to delaying the cracking of the base [20]. The addition of rubber can improve the frost resistance [21,22,23]. The mechanical properties and water stability of rubber powder cement-stabilized macadam base mixtures with different dosages were tested. When the mixing amount of rubber powder is not more than 8%, the water invasion resistance of the rubber powder cement-stabilized base coarse material is slightly lower than that of the benchmark cement-stabilized macadam base coarse material, and the scouring resistance is slightly improved [24,25,26]. Reasonable rubber powder content can improve the strength, stiffness, and crack resistance of the mixture [27]. When the dosage of retarder is appropriate, the hydration time of cement can be effectively delayed and the phenomenon of heat release concentration of cement hydration can be alleviated [28,29,30].

To sum up, many scholars have studied the application of rubber waste, but there are few studies on adding rubber powder and retarder into cement-stabilized macadam semi-rigid base at the same time. In order to improve the crack resistance and integrity of cement-stabilized macadam semi-rigid base, the mechanical properties and fatigue properties of rubber powder and retarder were studied by unconfined compressive strength test, splitting strength test, flexural tensile strength test, crack resistance test, and frost resistance test. Through industrial CT and scanning electron microscopy, the mechanism of action between the components was revealed from the microscopic point of view, and the improvement effect of compound doping on the road performance of semi-rigid base materials was further analyzed. In this way, the construction period can be shortened, and the double-layer paving of semi-rigid base can be realized. The lower semi-rigid base material is subjected to secondary rubbing and rolling, and the compactness can be improved. At the same time, the problem of poor crack resistance and frost resistance of semi-rigid base can be solved, and the application prospect is broad.

## 2. Materials and Methods

### 2.1. Aggregate

The natural aggregate used was from the Zhengzhou Municipal Engineering Corporation, produced in Yanshi, Luoyang. It was mainly limestone, and 4.75 mm was used as the dividing sieve of coarse and fine aggregate. The nominal maximum particle size of coarse aggregate was 19 mm. In the cement-stabilized macadam mixture, the crushed stone with certain strength, dry without weathering, hard, and wear-resistant was selected. The physical properties of coarse and fine aggregates are shown in Table 1.

### 2.2. Cement

The cement used was PO 42.5 (abbreviation of ordinary Portland cement with strength grade of 42.5 MPa) cement produced by Zhengzhou Tianrui Cement company Ltd. (Ruzhou, China). As the cement is used for the road base, the performance of the cement was tested according to the relevant test methods in the test code for cement and cement concrete of highway engineering jtge30-2005. The test results are shown in Table 2.

### 2.3. Powder

**Figure 1:**
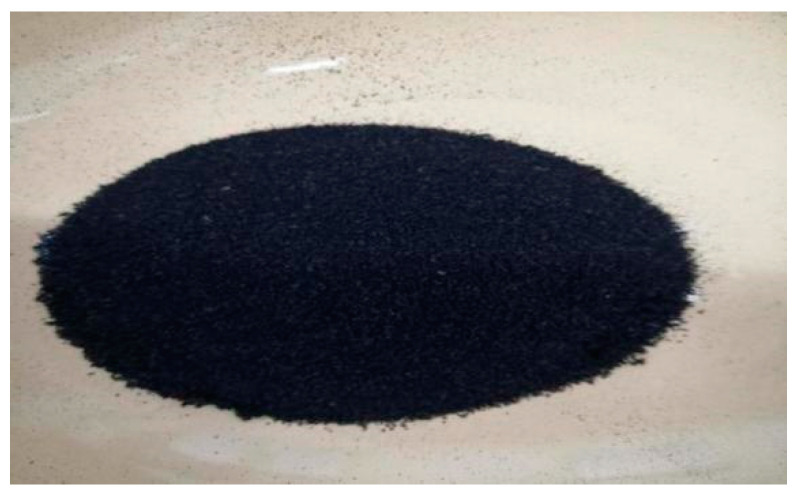
Rubber powder.

The rubber powder with mesh of 40 was used in this experiment, and the manufacturer was from Aorong Chemical New Materials Company Ltd., Changzhou, China. The density was 1.13 g/cm^3^, and the moisture content was 0.57%, as shown in Figure 1.

**Figure 2:**
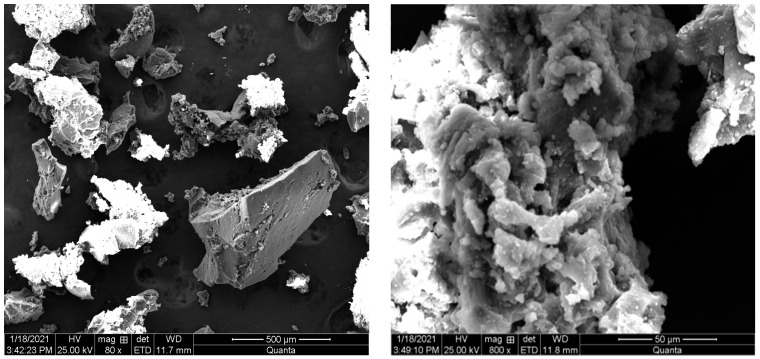
Micromorphology of rubber powder.

The morphology of 40 mesh rubber powder was analyzed by scanning electron microscopy, and the magnification was 80 times and 800 times, respectively. The electron microscope images are shown in Figure 2.

It can be seen from the electron microscope diagram that considering the morphology of the powder from the microscopic point of view, the surface of the powder was relatively rough and the distribution pattern was irregular. The particle size was about 410 μm. After 800 times amplification, it could be seen that there were a large number of small pores in the powder, which gives the powder better deformation properties.

### 2.4. Retarder Water

**Figure 3:**
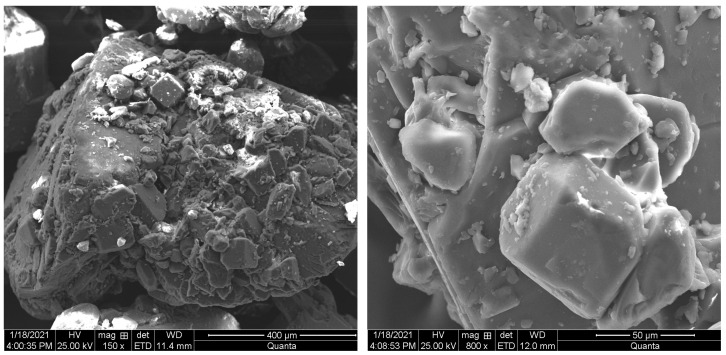
Microscopic morphology of retarder.

The retarder used was sodium gluconate produced by Tianjin Comiou Chemical Reagent Company Ltd. (Tianjin, China). It is a white crystalline powder, soluble in water, and has a relative molecular mass of 218.14. Sodium gluconate was scanned by electron microscope with magnifications of 150 and 800 times. Scanning electron microscope images are shown in Figure 3.

### 2.5. Water

The test water used was drinking water for residents from the Zhengzhou Water Supply Company. The water is used to mix cement-stabilized macadam mixture and molding and for maintenance of cement gel sand specimens.

### 2.6. Design of Test Blending Ratio

We selected the grading C-B-2 that could be used as the base course of expressway and class I highway and used the grading median to configure the mixture. When the cement dosage is more than 5% in the water-stabilized base, its ability to resist cracks deteriorate. Considering that the cement-stabilized macadam mixture needs a certain strength, 5% cement dosage is commonly used in engineering, so the cement dosage was 5%. The 40 mesh rubber powder was replaced by 0%, 0.5%, 1%, and 1.5% of natural fine aggregate with particle sizes of 0.15–0.3 mm. At the same time, a total of 16 mixing ratios were added with inorganic binders of 0%, 0.03%, 0.06%, and 0.09%. The specific design is shown in Table 3.

### 2.7. Test Method of Unconfined Compressive Strength

#### 2.7.1. Making and Health Preservation of Specimens

**Figure 4:**
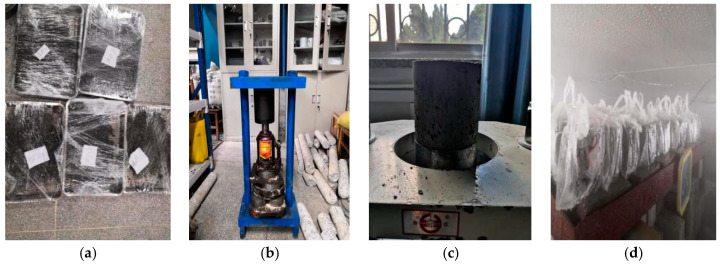
Specimen-making for unconfined compressive strength test: (**a**) Stuffy material; (**b**) Static pressure; (**c**) Demolding; (**d**) Curing.

The nominal maximum particle size of natural aggregate used in this section was not greater than 19.0 mm, which belongs to medium-grained soil. According to the relevant provisions of the specification, the cement-stabilized medium-grained soil mixture for indoor unconfined compressive strength test should be made into a cylindrical specimen with a diameter–height ratio of 1:1 and a size of 100 mm × 100 mm. In order to ensure the accuracy of the test results and reduce the influence caused by large data dispersion, at least nine specimens should be prepared, with a total of 16 × 9 × 3 = 432 unconfined compressive specimens. The process of specimen production is shown in Figure 4, and the relevant methods refer to the provisions of ‘inorganic binder test regulations’ T 0843-2009.

#### 2.7.2. Unconfined Compressive Test

**Figure 5:**
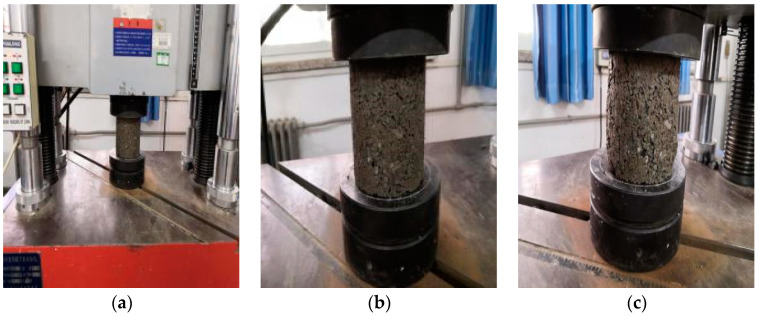
Unconfined compressive strength test: (**a**) Test preparation; (**b**) In test; (**c**) After test.

The test process is shown in Figure 5. According to the test method of ‘Test Specification for Inorganic Bond Stabilized Materials in Highway Engineering’ (JTG E51-2009), the unconfined compressive strength test of specimens cured to the corresponding age was carried out. The specimen was taken out from the iron frame in the curing room the day before the test was carried out and saturated in the flume for 24 h to ensure that the water flooded over the surface of the specimen 50 mm. After taking out, the moisture on the surface of the specimen was absorbed and the quality was weighed. The upper and lower surfaces of the specimens were cleaned and placed on a flat ball bearing test machine. Then, a 600 kN microcomputer-controlled electro-hydraulic servo testing machine (WAW-600B) and displacement-controlled loading mode were adopted. The loading rate was 1 mm/min, which is consistent with the specification requirements.

### 2.8. Splitting Strength Test

**Figure 6:**
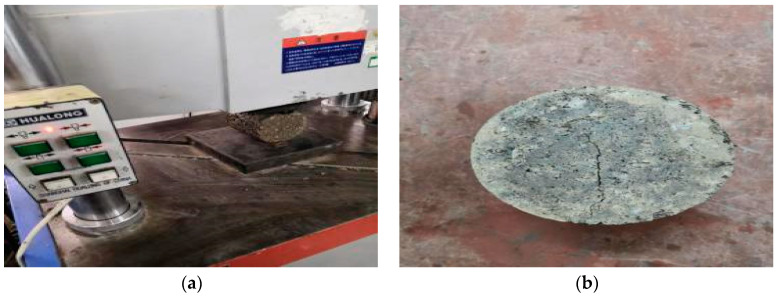
Splitting strength test: (**a**) In test; (**b**) After test.

Splitting strength tests of mixture specimens with different mass of rubber powder and retarder were carried out. The specimen was cylindrical with diameter × height = 100 mm × 100 mm. The fabrication and maintenance process were consistent with the unconfined strength specimen. A 600 kN microcomputer-controlled electro-hydraulic servo testing machine (WAW-600B) and displacement-controlled loading mode were adopted. The loading rate was 1 mm/min. The number of specimens in the same group was no less than 9, and the water was soaked for 24 h one day before the test. The time splitting test process of 90 d age mixture is shown in Figure 6.

### 2.9. Flexural Tensile Strength Test

**Figure 7:**
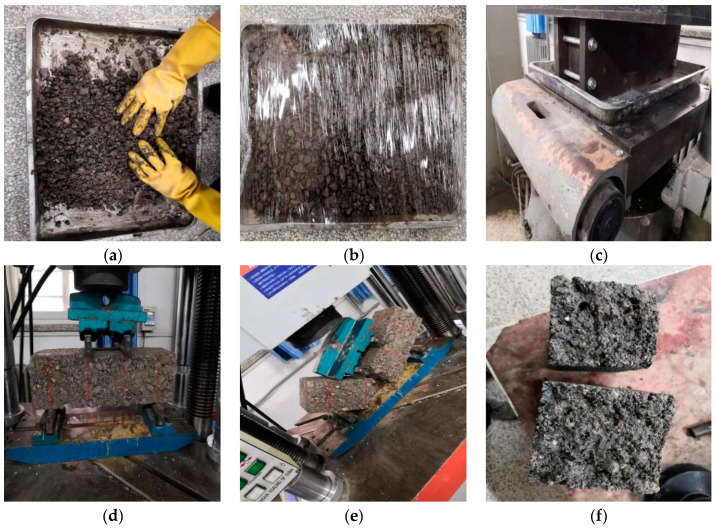
Flexural tensile strength test: (**a**) Mix; (**b**) Stuffy material; (**c**) Static pressure; (**d**) Test preparation; (**e**) In test; (**f**) After test.

The mixture specimens with different mass rubber powders and retarders were molded. The specimen size was wide × high × long = 100 mm × 100 mm × 400 mm, the concrete process is shown in Figure 7. The curing process was consistent with the cylindrical specimen, a total of 16 × 9 = 144 specimens. Curing time was 90 days, the day before the test, we carried out immersion for 24 h. A 600 kN microcomputer-controlled electro-hydraulic servo testing machine (WAW-600 B) was used, and uniform and continuous loading was maintained. The flexural test process is shown in Figure 7.

### 2.10. Dry Shrinkage Test

The specimen size required for the test was 100 mm × 100 mm × 400 mm, and the forming and curing process was consistent with the flexural and tensile test. The curing time was 7 days, and a total of 6 × 3 = 18 beams were needed.

**Figure 8:**
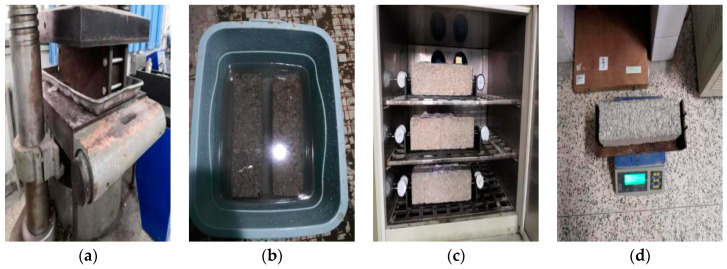
Specimen-making and observation of dry shrinkage test: (**a**) Forming; (**b**) Immersion; (**c**) Dry shrinkage measurement; (**d**) Water loss measurement.

The test method of this paper was used to determine the shrinkage deformation value of the specimen by the dial indicator displacement method. Some test procedures are shown in Figure 8.

### 2.11. Freeze-Thaw Performance Test

According to the test method T 0858-2009 recommended in the ‘Test Specification for Inorganic Binder Stabilized Materials for Highway Engineering’ (JTG E51-2009), the slow freezing air water thawing method was adopted in this paper. This test method is closer to the actual situation of repeated freezing and thawing in a wet environment in a seasonal frozen area.

**Figure 9:**
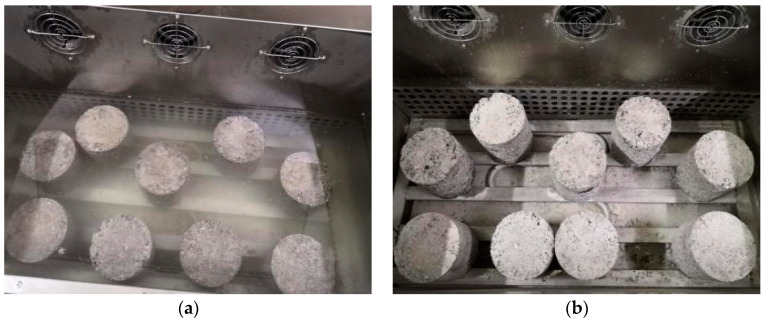
Freezing and thawing process: (**a**) Fused specimen; (**b**) Frozen test piece.

The freezing and thawing test was carried out by using the test method of standard maintenance for 28 d and freezing and thawing cycle five times. It was required that 18 standard specimens with Φ150 × h150 mm be prepared for each ratio, of which 9 were freeze-thaw specimens and 9 were comparative specimens without freeze-thaw tests. The instrument is LCD-18 automatic low temperature freeze-thaw testing machine produced by Tianjin Port Source Test Instrument Factory. The test process is shown in Figure 9.

### 2.12. Scanning Electron Microscope

**Figure 10:**
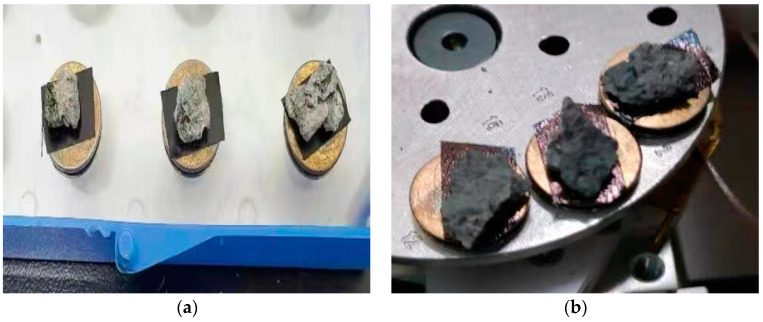
Operation flow of SEM: (**a**) Sample preparation; (**b**) Gold spray coating.

The sample was processed into 1 cm × 1 cm × 1 cm samples according to the size requirements of the carrier by scanning electron microscope. The sample was evenly placed on the sample plate by conductive tape. The vacuum gold plating was carried out in the coating instrument, and then moved to the scanning electron microscope for morphology observation. The instrument was mainly used to characterize the morphology by imaging the sample surface on the micro nano scale, focusing on observing the morphology of raw materials, the distribution of defects such as internal voids and micro cracks before and after mixture modification, the structural morphology of interface transition zone, and the quantity and growth morphology of cement hydration products. The sample is shown in Figure 10.

### 2.13. Industrial CT Test

The scanning imaging principle of industrial CT is as follows: the X-ray emitted by the ray source penetrates the detected object, and its energy will be scattered or partially absorbed by the detected object, resulting in the attenuation of the ray energy. The projection calculation is realized by using a specific algorithm. In this paper, with the help of industrial CT technology, R_0_C_0_, R_1_C_0_, and R_1_C_0.09_ groups of mixture specimens with different mixing ratios were scanned by CT, and the internal porosity, pore size, and quantity of the formed specimens were analyzed and counted.

## 3. Results and Discussion

### 3.1. Analysis of Unconfined Compressive Strength

#### 3.1.1. Influence of Rubber Powder Content on Unconfined Compressive Strength

**Figure 11:**
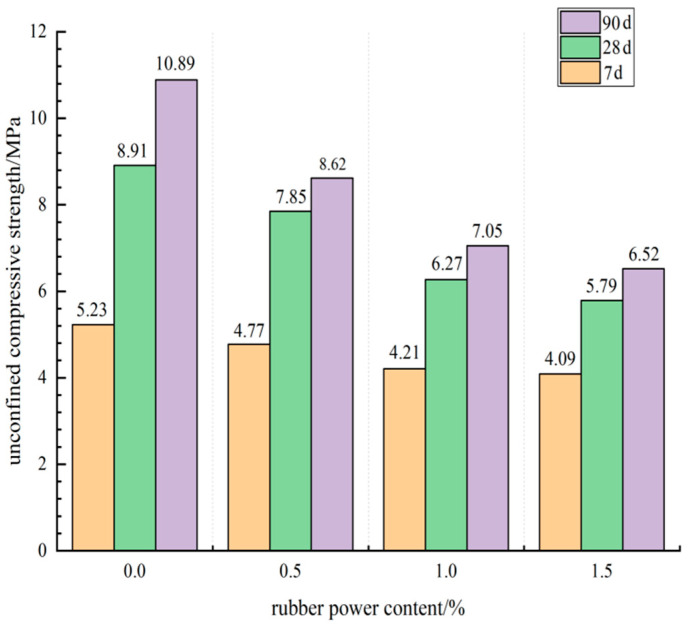
Effect curve of rubber powder content on unconfined compressive strength.

When the cement content of retarding composite cement-stabilized macadam base mixture with rubber powder was 5% and the sodium gluconate content was 0.06%, the influence of rubber powder mass content on the unconfined compressive strength of specimens at different ages is shown in Figure 11.

It can be seen from Figure 11 that when the cement content and sodium gluconate content were certain, with the increase of the mass fraction of rubber powder in cement-stabilized macadam mixture, the unconfined compressive strength of water-stabilized macadam mixture decreased gradually at 7 days, 28 days, and 90 days. When the rubber powder content was 1.5%, the strength loss of cement-stabilized macadam mixture specimen was more obvious. For example, when the cement content was 5% and 0.06% sodium gluconate was added, the 7-day compressive strength of the specimen without rubber powder reached 5.23 MPa. When the mass content of rubber powder reached 1.5%, the 7-day compressive strength of the specimen only reached 4.09 MPa, which was 21.8% lower than that of the blank group without rubber powder. From the 28 d compressive strength results, the compressive strength of specimens without rubber powder reached 8.91 MPa. When the mass content of rubber powder reached 1.5%, the compressive strength of specimens only reached 5.79 MPa. Compared with the blank group specimens without rubber powder, the strength decreased by 35.0%. From the analysis of 90 d compressive strength results, the compressive strength of the blank group specimen reached 10.89 MPa. When the mass content of rubber powder reached 1% and 1.5%, the compressive strength of the specimen reached 7.05 MPa and 6.52 MPa, and the strength decreased by 35.3% and 40.1%, respectively. The strength loss was more obvious.

#### 3.1.2. Effect of Retarder Dosage on Unconfined Compressive Strength

**Figure 12:**
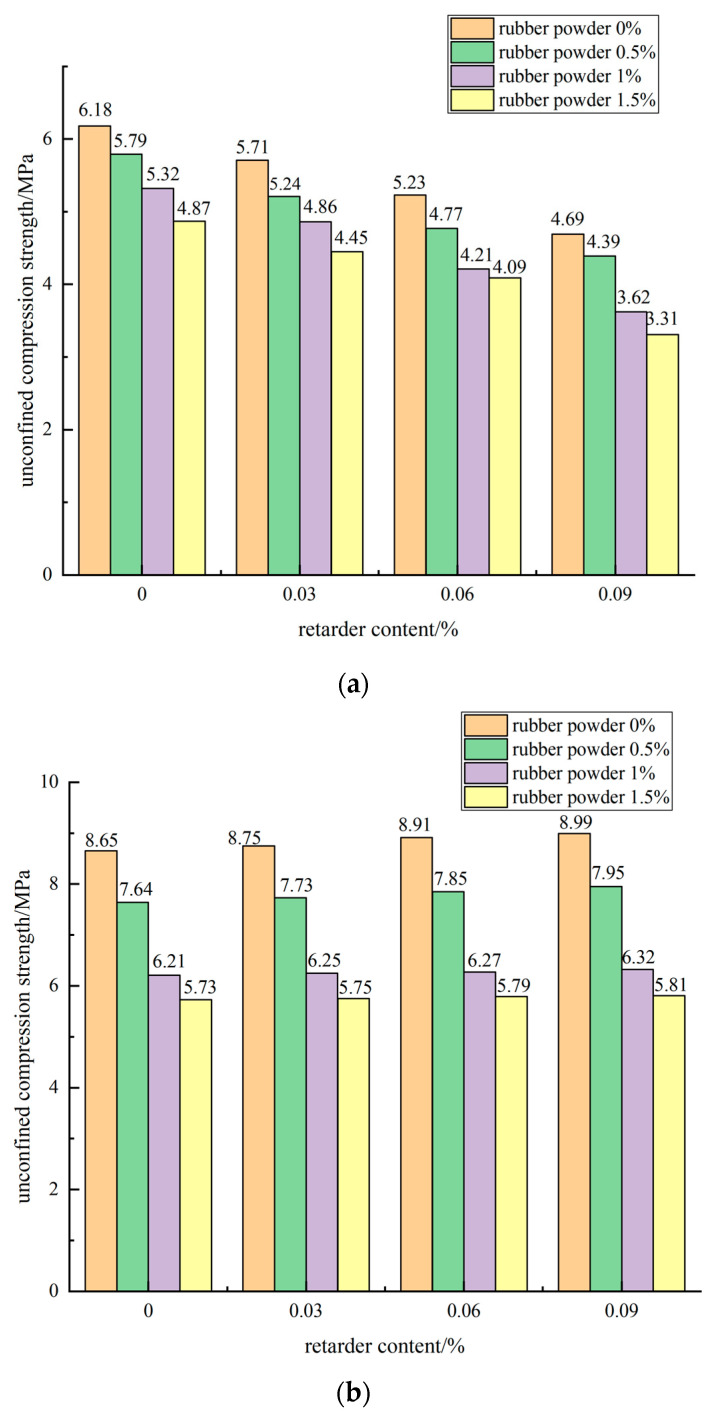

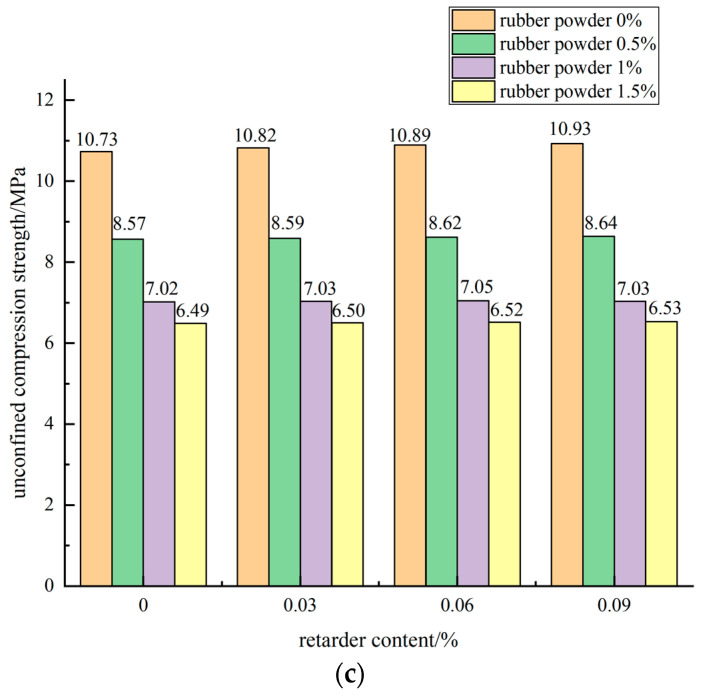
The influence curve of retarder content on the unconfined compressive strength of 7 days (**a**), 28 days (**b**), and 90 days (**c**).

When the cement dosage was 5% and the mass content of rubber powder was different, the influence of retarder dosage on the unconfined compressive strength of 7 days, 28 days, and 90 days is shown in Figure 12.

As shown in Figure 12a–c, the analysis of cement dosage of 5%, rubber powder dosage of 0.5%, and retarder dosage of different specimen compressive strength results shows that the retarder dosage of 0%, 0.03%, 0.06%, 0.09% specimens at 7 d compressive strength is 5.79 MPa, 5.24 MPa, 4.77 MPa, and 4.39 MPa, and the strength loss is 9.5%, 17.6%, and 24.2%. The 28 d compressive strength increased by 1.2%, 2.7%, and 4.1%, and the 90 d compressive strength increased to 0.2%, 0.6%, and 0.8%. When the dosage of rubber powder was 1.5%, the dosage of retarder was 0.09%, the strength loss of 7 d was more obvious, reaching 32.0%, and the strength of 28 d increased by 1.4%. The compressive strength of 90 d was only 0.1% higher than that of the blank group without retarder, and the increase was small. It can be seen that the addition of retarder had a negative impact on the early compressive strength of the mixture. With the increase of dosage, the strength of the mixture specimen decreased more obviously. The compressive strength of the mixture was slightly improved in the middle and late stages. The improvement of the compressive strength of 28 d was more obvious, and the strength of 90 d had little effect. Sodium gluconate had good retarding effect and slightly improved the strength of the mixture in the middle and late stages. Its delayed coagulation mechanism is mainly to inhibit the formation of cement hydration products, so as to achieve the purpose of retarding. There was no obvious change and influence on the types of cement hydration products.

#### 3.1.3. Effect of Composite of Rubber Powder and Retarder on Unconfined Compressive Strength

From the above analysis, it can be concluded that the addition of rubber powder and retarder reduce the early compressive strength of the mixture. Compared with the mixture with single rubber powder and retarder, the strength of the mixture specimen decreased obviously, and the compressive strength of the mixture in the middle and late stages were slightly improved. The unconfined compressive strength of the specimen for 7 d was analyzed. When the content of retarder was 0.03%, the strength of the cement stabilized mixture specimen with 0.5% and 1% rubber powder was 5.24 MPa and 4.86 MPa, respectively, which decreased by 7.3%. When the content of rubber powder was 0.5%, the strength of the cement stabilized mixture specimen with 0.03% and 0.06% retarder was 5.24 MPa and 4.77 MPa, respectively, which decreased by 9.0%. The compressive strength results of 28 d were analyzed, and the strength of specimens with the same dosage of rubber powder and retarder decreased by 19.1% and increased by 1.6%, respectively. The results of the 90 d analysis showed that the strength of specimens in the same dosage group decreased by 18.2% and increased by 0.3%, respectively. It can be seen that the increase of the amount of rubber powder reduced the compressive strength of the mixture in the middle and late stages, and the increase of the amount of retarder improved the compressive strength of the mixture in the middle and late stages, because the rubber powder absorbed part of the stress when the specimen was under pressure, but its easy deformation made it easy to separate from the aggregate of the mixture and reduce the mechanical properties. The retarder promoted the hydration of cement and generated more gels, which were evenly distributed in the mixture. It strengthened the connection between rubber powder and other aggregates and improved the mechanical properties.

### 3.2. Splitting Strength Analysis

#### 3.2.1. Effect of Rubber Powder Content on Splitting Strength

**Figure 13:**
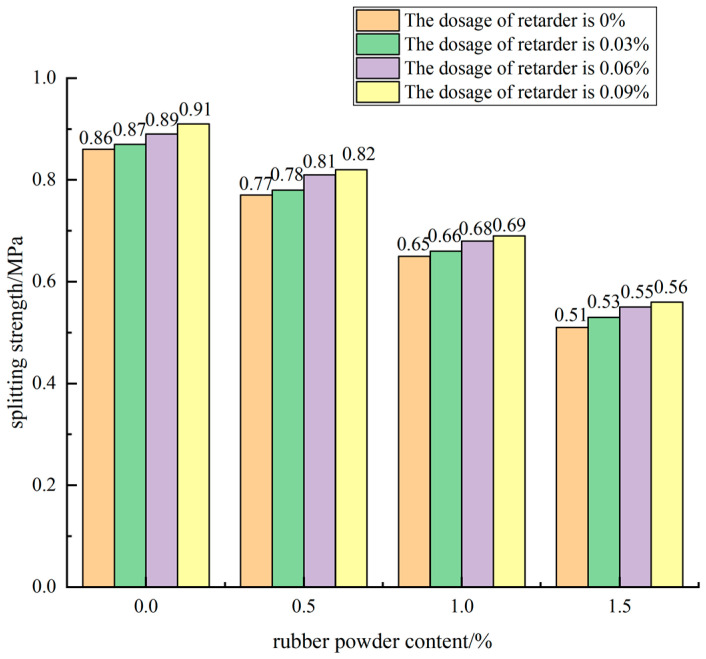
Effect curve of rubber powder content on splitting strength.

When the cement dosage was 5% and the retarder dosage was different, the influence of the rubber powder content on the splitting strength is shown in Figure 13.

It can be seen from Figure 13 that the test data with cement dosage of 5% and retarder dosage of 0.06% were selected for analysis. The splitting strength of the blank group without rubber powder reached 0.89 MPa; when the rubber powder content was 0.5% and 1%, the splitting strength was 0.81 MPa and 0.68 MPa, respectively. Compared with the blank group without rubber powder, the strength decreased by 9.0% and 23.6%. When the rubber powder content reached 1.5%, the splitting strength was only 0.55 MPa, which was 38.2% lower than that of the specimen without rubber powder. Therefore, adding rubber powder to cement-stabilized macadam mixture had a negative impact on the splitting strength of the specimen, and with the increase of rubber powder content, the loss of 90 d splitting strength of water-stabilized macadam mixture was more serious.

#### 3.2.2. Effect of Retarder Dosage on Splitting Strength

**Figure 14:**
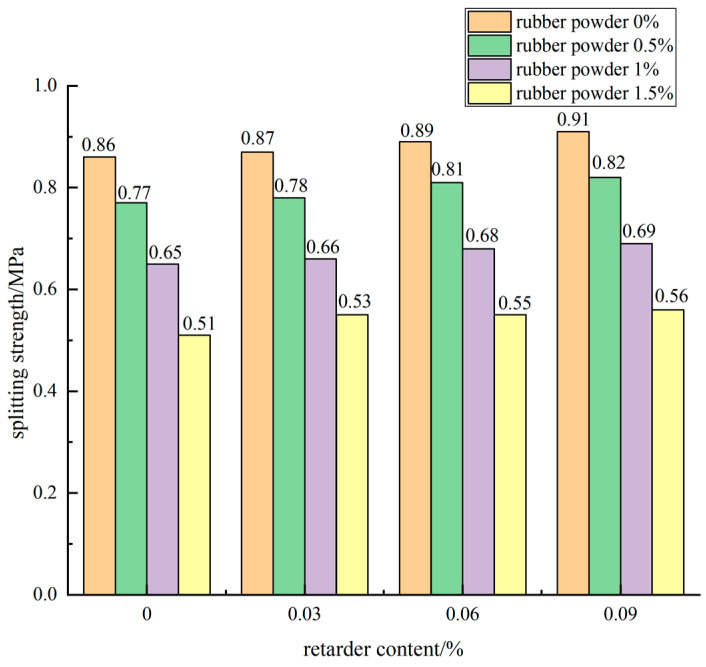
Effect curve of retarder content on splitting strength.

The influence of the increase of retarder dosage on the splitting strength when the cement dosage was 5% and the dosage of rubber powder was different is shown in Figure 14.

It can be seen from the diagram that with the increase of retarder content, the splitting strength of cement-stabilized macadam mixture was improved, but the increase of the curve was small. When the cement content was 5% and the rubber powder content was 0.5%, the 90-day splitting strength of the blank control group without retarder was 0.77 MPa. When the retarder content was 0.03%, 0.06%, and 0.09%, the splitting strength of the mixture reached 0.78 MPa, 0.81 MPa, and 0.82 MPa, which was 2.3%, 5.2%, and 6.5% higher than that of the blank group without retarder.

#### 3.2.3. Effect of Compound of Rubber Powder and Retarder on Splitting Strength

When the dosage of rubber powder was 0.5% and the dosage of retarder was 0.03%, the 90 d splitting strength of the mixture specimen was 0.78 MPa, the dosage increased to 0.06% and 0.09%, and the splitting strength of the specimen was 0.81 MPa and 0.82 MPa, respectively. Compared with 3.8% and 5.1%, the splitting strength of the mixture increased by 3.0% and 4.5% when the dosage of rubber powder was 1%. When the content of retarder was 0.03%, the content of rubber powder was 1% and 1.5%, respectively, compared with 0.5%, the splitting strength of the mixture specimen decreased by 15.4% and 32.1%, respectively. When the content of retarder was 0.06%, the strength decreased by 16.0% and 32.1%, respectively.

According to the above analysis, the splitting strength of the mixture decreased with the increase of the content of rubber powder, and slightly increased with the increase of the content of retarder. Because the retarder was added to improve the uniformity of the cement mortar, the cement mortar was equivalent to “lubricant” to wrap the rubber powder and aggregate and reduce the mutual friction, so that the smaller aggregate could fill in the macropores, reduce the internal porosity, reduce the generation of through cracks, and improve the strength of the mixture.

### 3.3. Flexural Tensile Strength Analysis

#### 3.3.1. Effect of Rubber Powder Content on Flexural Tensile Strength

**Figure 15:**
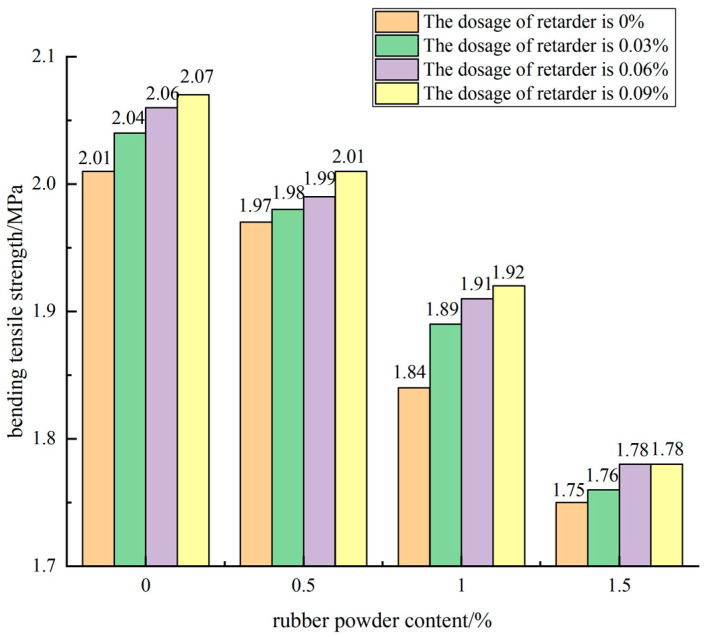
Effect curve of rubber powder content on flexural tensile strength.

The influence of rubber powder dosage on the 90 d flexural tensile strength of the mixture when cement dosage was 5% and retarder dosage was different is shown in Figure 15.

It can be seen from Figure 15 that the addition of rubber powder had a certain reduction effect on the 90 d flexural tensile strength of cement-stabilized mixture. With the increase of rubber powder content, the decrease of flexural tensile strength of cement-stabilized mixture was more obvious. Taking the retarder content of 0.09% as an example, the flexural tensile strength of the cement stabilized mixture specimen without rubber powder for 90 d was 2.07 MPa, and the flexural tensile strength of the specimen with 0.5% and 1% rubber powder was 2.01 MPa and 1.92 MPa, which were reduced by 2.9% and 7.2%. When the rubber powder content reached 1.5%, the flexural tensile strength of the cement stabilized mixture was only 1.84 MPa, which was 11.1% lower than that of the specimen without rubber powder, and the decrease was 3.8 times that of the rubber powder content of 0.5%.

#### 3.3.2. Effect of Retarder Content on Flexural Tensile Strength

**Figure 16:**
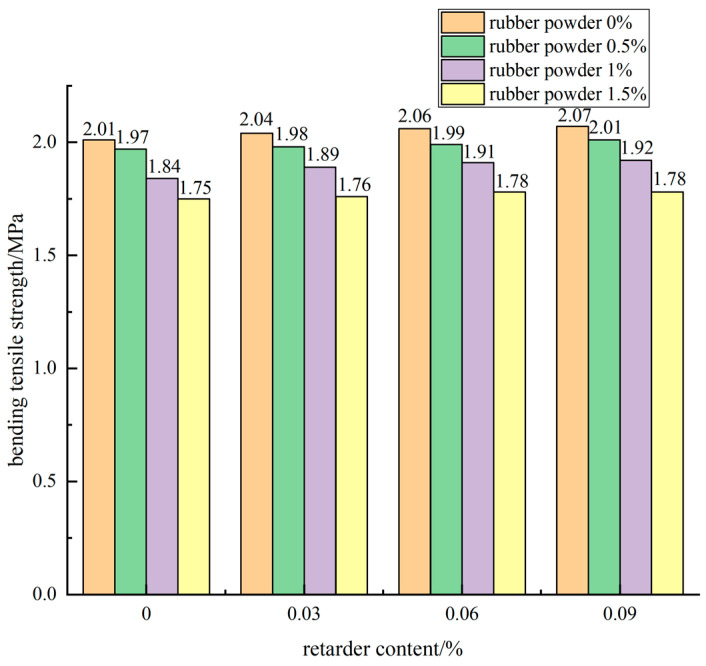
Effect curve of retarder content on flexural tensile strength.

The influence of retarder dosage on the 90 d flexural tensile strength of the mixture when the cement dosage was 5% and the rubber powder dosage was different is shown in Figure 16.

It can be seen from Figure 16 that the addition of retarder had an enhanced effect on the 90 d flexural tensile strength of cement-stabilized mixture. With the increase of the content of retarder, the flexural tensile strength of cement stabilized mixture was improved, but the improvement effect was not obvious. Taking the rubber powder content of 0.5% as an example, the flexural tensile strength of the cement stabilized mixture specimen without retarder for 90 d was 1.97 MPa, and the flexural tensile strength of the specimen with retarder content of 0.03%, 0.06%, and 0.09% was 1.98 MPa, 1.99 MPa, and 2.01 MPa, respectively, which is slightly higher than that of the specimen without retarder.

#### 3.3.3. Effect of Compound of Rubber Powder and Retarder on Flexural Strength

From the above analysis, it can be concluded that the flexural tensile strength of retarding composite semi-rigid cement-stabilized mixture specimens with rubber powder decreased with the increase of rubber powder content and increased with the increase of retarder content. Taking 0.5% rubber powder content as an example, when the dosage of retarder was 0.03%, 0.06%, and 0.09%, the flexural tensile strength of the specimen was 1.98 MPa, 1.99 MPa, and 2.01 MPa, respectively, which was increased by 0.5% and 1.5% compared with that of the control. When the dosage of retarder was increased to 1%, the flexural tensile strength of the mixture was increased by 1.1% and 1.6%, respectively. The retarder improved the flexural tensile strength of the mixture specimen, but the increase was small. When the content of retarder was 0.03%, the content of rubber powder was 0.5%, 1%, 1.5%, and the flexural tensile strength of the mixture specimen decreased by 4.5%, 11.1%, respectively. When the content of retarder was 0.06%, the strength decreased by 4.0%, 10.6%, respectively. Rubber powder has a great impact on the flexural tensile strength of the mixture specimen.

### 3.4. Dry Shrinkage Performance Analysis

**Figure 17:**
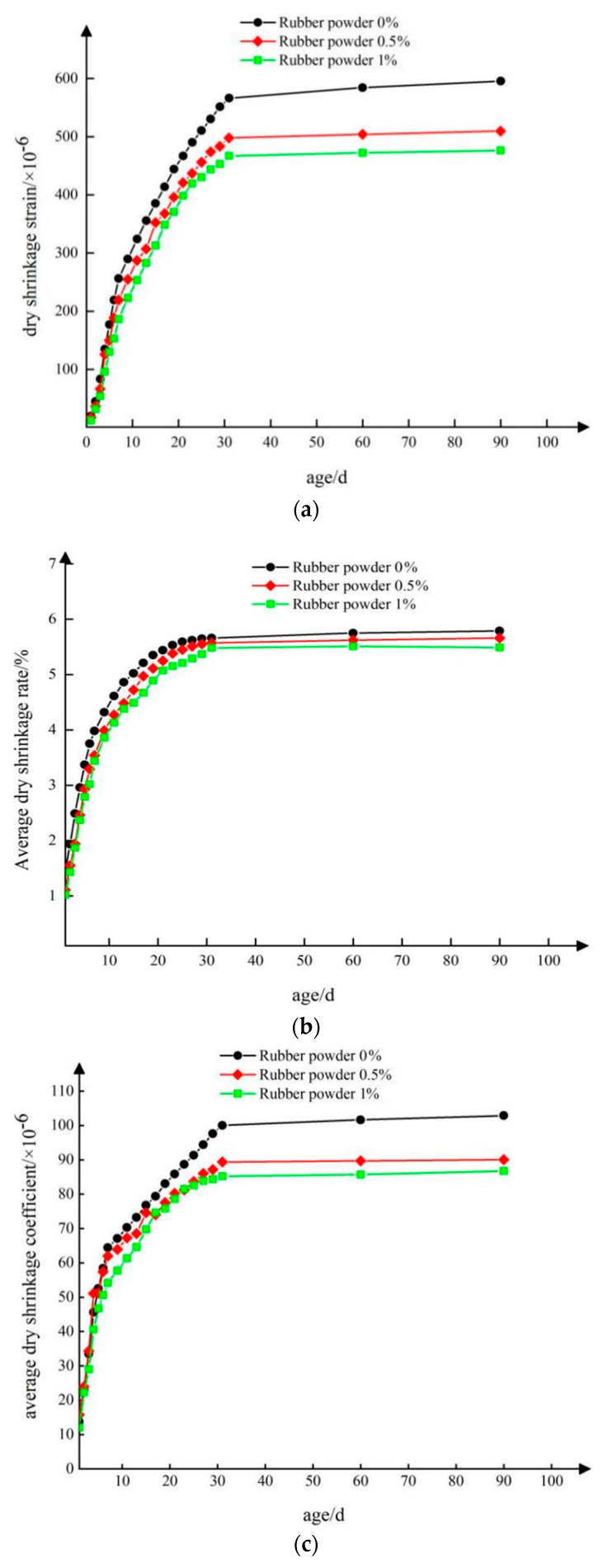
Curve diagram of relationship between dry shrinkage strain (**a**), average water loss rate (**b**), average dry shrinkage coefficient (**c**), and age.

Figure 17a–c shows the relationship between the total amount of dry shrinkage strain, average water loss rate, and average dry shrinkage coefficient of the test piece and the test piece when the retarder content was 0.06%.

It can be seen from Figure 17a that the dry shrinkage strain of the retarded composite semi-rigid base mixture mixed with rubber powder in the test to 7 days, the cumulative dry shrinkage strain of the three groups of specimens with 0%, 0.5%, and 1% rubber powder content reached 43.04%, 43.05%, and 39.13%, respectively, and the cumulative dry shrinkage strain of the three groups of specimens reached 95.05%, 97.11%, and 98.05%, respectively at 31 days, which were higher than 95%. This shows that the dry shrinkage deformation of the cement stabilized mixture mainly occurs in the early maintenance of the specimen. With the increase of the maintenance age, the dry shrinkage deformation of the mixture almost stopped. It can be seen that the addition of rubber powder in the early maintenance of the specimen had a favorable impact on its dry shrinkage strain, that is, it reduced the dry shrinkage strain of the cement stabilized mixture.

From Figure 17b, the relationship between the average water loss rate and the test age shows that the variation trend of the average water loss rate was similar to the variation curve of the dry shrinkage strain with the age. The average water loss rate of the cement stabilized mixture increased with the increase of time. The water loss of the specimen in the early stage increased first, and the growth rate slowed down and tended to be stable in the late stage. With the increase of rubber powder content, the water loss rate of cement stabilized mixture was lower than that of water-stabilized mixture without rubber powder. Rubber powder can reduce the water loss rate of water stabilized mixture to a certain extent.

It can be seen from Figure 17c that with the increase of rubber powder content, the average dry shrinkage coefficient of the mixture showed a downward trend. In the early stage of water loss of the specimen, the average dry shrinkage coefficient of the specimen with 1% rubber powder was significantly smaller than that of the other two groups of specimens, which indicates that the water loss and dry shrinkage sensitivity of the base material decreased after the addition of rubber powder, which is conducive to reducing the early dry shrinkage cracking of the cement stabilized mixture.

The comprehensive comparative analysis of the three basic indexes of the above drying shrinkage test showed that the drying shrinkage indexes of the mixture specimens with rubber powder were lower than those of the blank group, which shows that the rubber powder can reduce the drying shrinkage deformation of the cement-stabilized base mixture to a certain extent, mainly inhibiting the early water evaporation of the specimen, which is characterized by the decrease of the water loss rate, which is beneficial to the early crack resistance of the base.

### 3.5. Freeze-Thaw Performance Analysis

#### 3.5.1. Effect of Rubber Powder Content on Freeze-Thaw Performance

**Figure 18:**
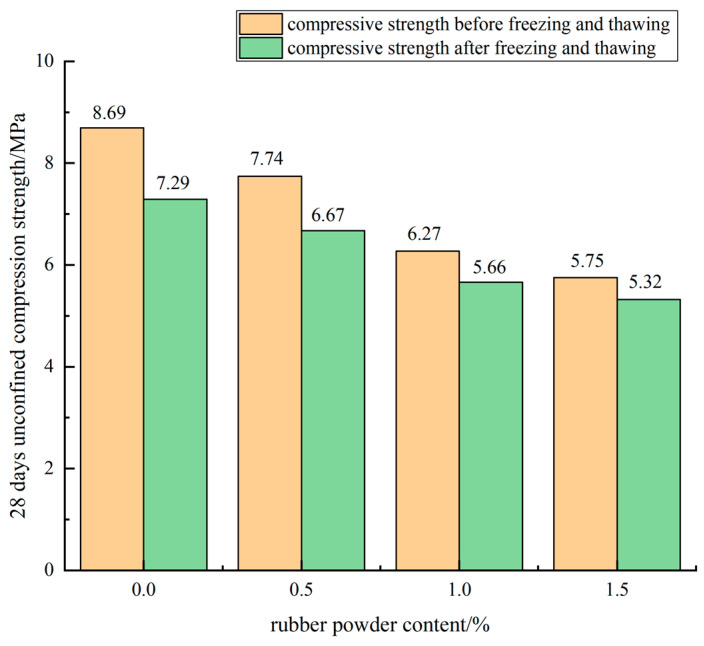
Influence curve of rubber powder content on BDR.

The influence curve of the rubber powder content on the frost resistance of the mixture specimen when the cement content was 5% and the retarder content was 0.03% is shown in Figure 18.

It can be seen from Figure 18 that the compressive strength of the cement-stabilized mixture specimen after five freeze-thaw cycles was lower than that of the specimen without freeze-thaw cycles. With the addition of rubber powder, the residual compressive strength of the specimen after freeze-thaw cycling was relatively high, indicating that the compressive strength of the mixture decreased slowly after frost heaving. When the mass of rubber powder was 0%, the BDR value of the 28 d specimens in this group was 83.5%, and when the rubber powder content was 0.5%, the BDR value of the 28 d cement stabilized mixture specimens was 86.2%, which was 2.7% higher than that of the specimens without rubber powder. When the rubber powder content reached 1%, the BDR value of the specimen was 90.3%, which was 6.8% higher than that of the blank control group. When the rubber powder content reached 1.5%, the BDR value of the specimen was 92.3%, which was 8.8% higher than that of the blank control group. According to the variation law of the BDR value of the specimen, the rubber powder could improve the frost resistance of the mixture. With the increase of the rubber powder content, the frost resistance of the cement-stabilized mixture improved more obviously.

#### 3.5.2. Effect of Retarder Dosage on Freeze-Thaw Performance

**Figure 19:**
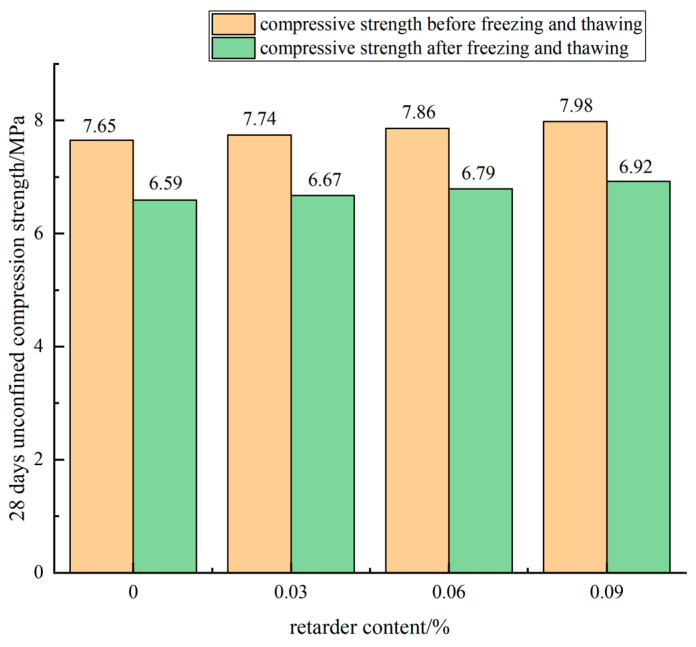
Influence curve of retarder content on BDR.

Figure 19 shows the cement content of 5% and the rubber powder content of 0.5% with the change of retarder content: the broken line diagram of compressive strength changes of 28 d cement stabilized mixture specimens before and after freezing and thawing.

It can be seen from Figure 19 that with the increase of retarder content, the compressive strength of the mixture specimen before and after freezing and thawing was improved, that is, the addition of retarder improved the frost resistance of the cement-stabilized mixture specimen to a certain extent. The unconfined compressive strength of the mixture without retarder was 6.59 MPa after 28 days of freeze-thaw cycle, and the compressive strength of the blank control group was 7.65 MPa. The BDR value of 28 days was 86.1%, and the strength loss was 13.9%. When the retarder content was 0.03%, 0.06%, and 0.09%, the strength of the specimens after freezing and thawing was 6.67 MPa, 6.79 MPa, and 6.92 MPa, respectively. The strength of the specimens in the control group was 7.74 MPa, 7.86 MPa, and 7.98 MPa, respectively. The BDR values of the cement-stabilized mixture after 28 days are 86.2%, 86.4%, and 86.7%, respectively. The BDR value increased and the strength loss is decreased. The unconfined compressive strength of the mixture specimens after freeze-thaw cycles was analyzed. With the increase of retarder content, the strength of the specimens after freeze-thaw was 6.59 MPa, 6.67 MPa, 6.79 MPa, and 6.92 MPa, respectively. Compared with the blank group specimens, the strength was increased by 1.2%, 3.0%, and 5.0%, respectively, indicating that retarder had an effect on improving the mixture.

#### 3.5.3. Effect of Compound of Rubber Powder and Retarder on Freeze-Thaw Performance

From the above analysis, it can be seen that when the rubber powder content was constant, the frost resistance of the mixture specimen was not significantly improved. When the coagulant content was constant, the frost resistance of the mixture specimen was greatly improved. Since the retarder can improve the compactness and integrity of the mixture, and improve the frost resistance of the mixture, the rubber powder filled in the mixture can also improve the compactness. When the mixture was subjected to freezing and thawing cycles, the rubber powder absorbed part of the stress, thereby reducing the damage to the internal mixture caused by frost heaving. The interaction between the two can effectively slow down the development of cracks and improve the frost resistance of the mixture.

### 3.6. Micro-Analysis

#### 3.6.1. Effect of Rubber Powder on Microstructure of Cement-Stabilized Mixture

**Figure 20:**
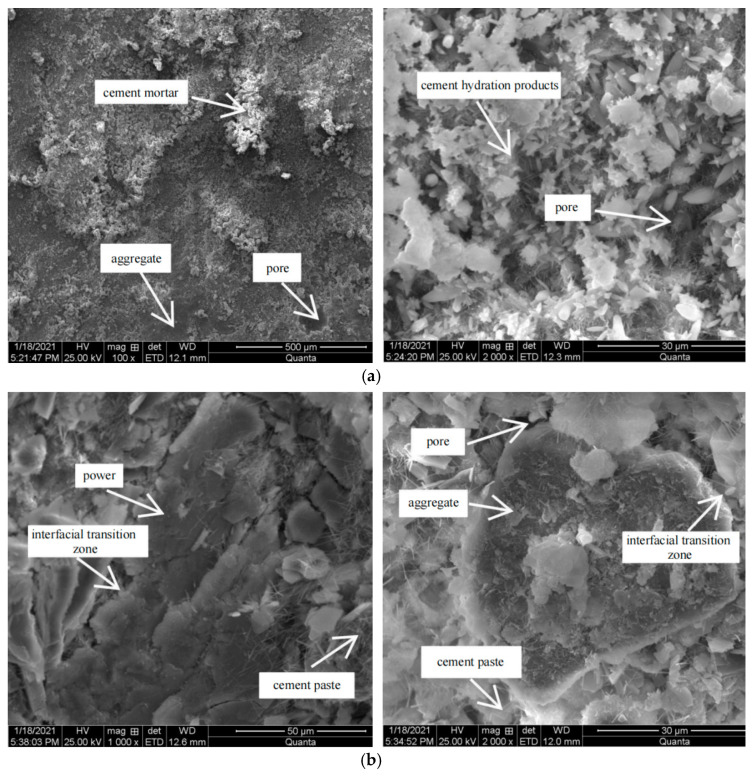
SEM images of R_0_C_0_ (**a**) and R_0.5_C_0_ (**b**) specimens.

In Figure 20a is the microscopic morphology of R_0_C_0_ group under scanning electron microscope, with magnifications of 100 and 2000 times. Figure 20b is the microscopic morphology of R_0.5_C_0_ group under scanning electron microscope, with magnifications of 500 and 2000 times.

It can be seen from Figure 20a that there were a large number of pores and microcracks with different sizes in the interfacial transition zone between cement mortar and aggregate, aggregate, and cement hydration products, which is the main reason for the poor road performance of cement-stabilized mixture. The cracks distributed in the interface transition zone and the micro cracks formed by cement hydration products account for a large proportion. There was a small amount of hydration crystal growth at the crack, but the small number cannot provide enough strength to fill the pores.

From Figure 20b, it can be seen that the mixture with rubber powder was denser than the mixture without rubber powder, and the aggregate could be well combined with the cement paste. However, the rubber powder could not be completely wrapped by the cement slurry, and some were exposed outside, that is, the interface transition zone of the two was not covered by the cement slurry, which led to the decrease of the bonding force of the internal parts and the integrity of the mixture and affected the mechanical properties. The cracks between cement hydration products in the sample were greatly reduced, which also explains that the addition of rubber powder reduced the pores existing in free water, reduced the expansion stress caused by external environment freezing to a certain extent, and reduced the channel of water loss. From a macro perspective, the incorporation of rubber powder improved the dry shrinkage performance and frost resistance of cement-stabilized mixture.

#### 3.6.2. Effect of Retarder on Microstructure of Cement-Stabilized Mixture

**Figure 21:**
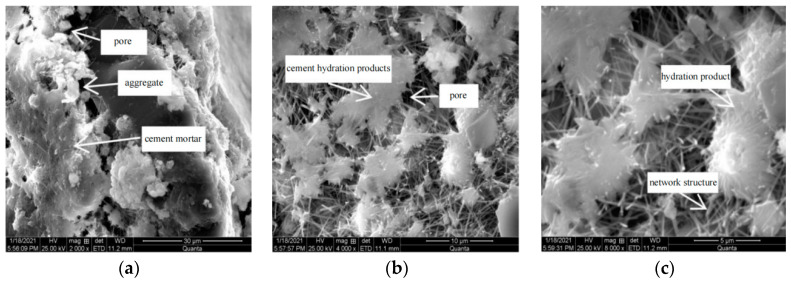
SEM images of R_0.5_C_0.09_ specimens were magnified 2000 times (**a**), 4000 times (**b**), 8000 times (**c**), respectively.

Figure 21 shows the microscopic morphology of R_0.5_C_0.09_ group under scanning electron microscope, with magnifications of 2000, 4000, and 8000 times, respectively.

It can be seen from Figure 21 that the cement paste coated on the aggregate surface was more uniform. Compared with the mixture without retarder, the interface transition boundary between aggregate and mortar was not obvious, and there was no large number of interface cracks. This did not cause the separation of the aggregate and the cement paste due to the poor adhesion of the interface transition zone when the structure was stressed, resulting in the reduction of the number of through cracks due to the poor synergy between the aggregate and the cementitious material, indirectly improving the compressive performance of the mixture. When the cement hydration products were enlarged by 4000 times, it was obviously seen that the flocculent C-S-H gel increased and the pores inside the gel decreased. When the cement hydration products were enlarged by 8000 times, the length of acicular AFt increased, and the interaction between crystals formed a complex network support structure. A large number of hydration products were filled in the pores, which can refine the capillary pores and dense micro pores. This shows that the addition of retarder had an impact on the appearance and morphological characteristics of cement hydration products. The formation of a large number of hydration products improved the integrity of the mixture, mainly manifested in the improvement of mechanical properties.

Based on the observation of the micro morphology of the above three groups of samples, it could be seen that the addition of rubber powder improved the distribution of internal pores in the mixture, and the cracks with large aperture were filled and refined into small cracks. The addition of retarder had a certain influence on the internal pores of the specimen, the interfacial transition zone, and the distribution of cement hydration products, so that the mechanical properties, dry shrinkage properties, and frost resistance of the cement-stabilized mixture were improved.

#### 3.6.3. Industrial CT Test Analysis

**Figure 22:**
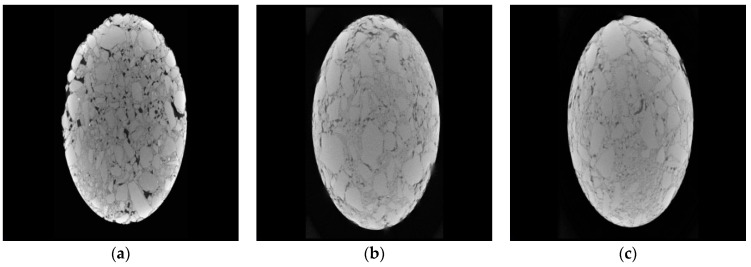
Three-dimensional CT scanning images of R_0_C_0_ (**a**), R_1_C_0_ (**b**) and R_1_C_0.09_ (**c**) mixtures.

Figure 22 is the three-dimensional CT scan diagram of R_0_C_0_, R_1_C_0_, and R_1_C_0.09_ mixture specimens with different blending ratios.

It can be seen from Figure 22 that the number of pores in the blank group without rubber powder and retarder is more, and there are more connected pores and semi connected pores, because the intercalation effect between aggregates is not obvious, and the specific surface area of coarse and fine aggregates in contact with each other is small; From the top view of the mixture specimen with only rubber powder, it can be seen that the number of large pore diameter of the specimen is significantly reduced, which is mainly related to the deformation performance of rubber powder. Rubber powder can be filled in the larger pores as a filler, thus reducing the porosity of the mixture specimen; From the top view of the mixture specimen mixed with rubber powder and retarder, it can be clearly seen that the proportion of white parts increases, the specific surface area of their mutual contact increases, and the pore distribution is mainly manifested in the uniform distribution of micro pores, which indicates that in addition to the pores with large diameter filled by rubber powder, the retarder increases the hydration products of cement, These hydration products can also play the role of filling, especially the acicular ettringite crystals interact to form a dense network structure, making the large-diameter pores further refined; In addition, more cement paste wrapping plays a "lubricating" effect, and the aggregates are closely embedded with each other, further reducing the porosity of the mixture. 

## 4. Conclusions

(1) Rubber powder had an adverse effect on the compressive strength, splitting strength, flexural tensile strength, and other mechanical properties of the mixture. With the increase of the amount of rubber powder, the mechanical properties of cement-stabilized macadam specimens decreased more obviously. When the content of rubber powder reached 1.5%, it decreased by 11.1% compared with the samples without rubber powder, and the decrease was 3.8 times as much as when the content of rubber powder was 0.5%.
(2) The addition of retarder could improve the mechanical properties such as compressive strength, splitting strength, and flexural tensile strength of cement-stabilized macadam mixture. When the content of retarder was 0.09%, the compressive strength, splitting strength, and flexural tensile strength increased by 1.9%, 6.5%, and 2.0%, respectively.
(3) The dry shrinkage strain, average water loss rate, and average dry shrinkage coefficient of the cement-stabilized mixture showed a decreasing trend with the increase of the rubber powder content. This is because the rubber powder was added to the mixture. As fine aggregate, it filled in the pores inside the specimen to improve the compactness of the mixture and refined the pores with large diameter into small and medium pores. The characteristics of large deformation can absorb part of the expansion stress generated during freezing and improve the dry shrinkage performance and frost resistance of the mixture.
(4) Retarder can improve the frost resistance of the mixture. Since the addition of sodium gluconate can promote the formation of cement hydration products, the filling of hydration products in the pores can reduce the porosity of the specimen, thereby improving the compactness of the specimen, reducing the free water channel inside the specimen, enhancing the bonding force between the interiors of the mixture, improving the residual ratio of freeze-thaw compressive strength of the water stabilized mixture, and improving its frost resistance.
(5) According to the microscopic analysis, the rubber powder is different from the natural aggregate and cannot be well combined with the cement slurry. The addition of retarder improved the integrity of the mixture, changed the morphological characteristics of the hydration products, formed a dense network structure, provided a certain strength, and then improved the mechanical properties and durability of the cement-stabilized macadam mixture.
(6) In practical engineering, both of them are mixed together. Considering that too much retarder content will affect the early strength production of semi-rigid base, and too much rubber powder content will affect the mechanical properties of semi-rigid base, it is suggested that the retarder content should be controlled at 0.03–0.06%, and the rubber powder content should be 0.5–1%.

## Figures and Tables

**Table 1:** Test results of physical properties of aggregates.

Performance Index		Bulk Density (g/cm^3^)	Apparent Density (g/cm^3^)	Water Absorption (%)	Crush Value (%)	Needle and Flake Content (%)	Mud Content (%)
coarse aggregate	10–20 (mm)	1.475	2.746	1.91	15.6	9.2	0.1
	5–10 (mm)	1.524	2.783	1.89	—	8.4	0.0
fine aggregate	3–5 (mm)	1.538	2.883	2.15	—	—	—
	0–3 (mm)	1.584	2.894	2.67	—	—	—

**Table 2:** Test results of cement performance.

**Test Items**		**Test Result**		**Specification Requirement**
fineness (F)		3.6%		≤10% (F: 0.08 mm square sieve percentage)
standard consistency water consumption (P)		25.7%		P: standard consistency water consumption
initial setting time		257 min		≥180 min
final setting time		374 min		≥360 min
**Test Items**		**Test Result**		**Specification Requirement**
stability		1.0 mm		≤5 mm
strength of cement mortar (MPa)	flexural strength	3 d	5.9	≥3.5 MPa
		28 d	8.7	≥6.5 MPa
	compressive strength	3 d	25.8	≥17.0 MPa
		28 d	48.7	≥42.5 MPa

**Table 3:** Mixing proportion design table of retarded semi-rigid base mixed with rubber powder.

Mixing Method No. of Rubber Powder and Retarder	Rubber Percentage (R)	Sodium Gluconate Percentage(C)
R_0_-C_0_	0%	0%
R_0_-C_0.03_	0%	0.03%
R_0_-C_0.06_	0%	0.06%
R_0_-C_0.09_	0%	0.09%
R_0.5_-C_0_	0.5%	0%
R_0.5_-C_0.03_	0.5%	0.03%
R_0.5_-C_0.06_	0.5%	0.06%
R_0.5_-C_0.09_	0.5%	0.09%
R_1_-C_0_	1%	0%
R_1_-C_0.03_	1%	0.03%
R_1_-C_0.06_	1%	0.06%
R_1_-C_0.09_	1%	0.09%
R_1.5_-C_0_	1.5%	0%
R_1.5_-C_0.03_	1.5%	0.03%
R_1.5_-C_0.06_	1.5%	0.06%
R_1.5_-C_0.09_	1.5%	0.09%

Note: R-C, R refers to the percentage of rubber powder in the mixture, and C refers to the percentage of sodium gluconate in the mixture.

## Data Availability

All data that support the findings of this study are included within the article (and any supplementary files).
